# Superiority of allo-SCT to auto-SCT in high-risk T-cell lymphoblastic lymphoma/leukemia patients with aaIPI ≥3: insights from a retrospective, multicenter analysis

**DOI:** 10.1097/BS9.0000000000000222

**Published:** 2025-04-11

**Authors:** Xiaodan Luo, Zhiping Fan, Runhui Zheng, Fen Huang, Na Xu, Pengfei Qin, Jingren Lin, Chunyan Wang, Huiqiang Huang, Huo Tan, Qifa Liu

**Affiliations:** a Hematology Department, The Fifth Affiliated Hospital of Guangzhou Medical University, Guangzhou, China; b Department of Hematology, Nanfang Hospital, Southern Medical University, Guangzhou, China; c Department of Hematology, The First Affiliated Hospital of Guangzhou Medical University, Guangzhou, China; d Department of Medical Oncology, State Key Laboratory of Oncology in South China/Cancer Center, Collaborative Innovation Center for Cancer Medicine, Sun Yat-sen University, Guangzhou, China

**Keywords:** Allogenic, GvHD, Lymphoma, T-cell, Transplantation

## Abstract

High recurrence is a major challenge in treating T-lymphoblastic lymphoma (T-LBL). Allogeneic (allo-) or autologous (auto-) stem cell transplantation (SCT) is recommended to reduce relapse, though the optimal choice remains unclear. This study retrospectively analyzed outcomes in T-LBL patients undergoing SCT, with 44 patients receiving allo-SCT and 25 receiving auto-SCT. After a median follow-up of 115 months, the 5-year cumulative incidence of relapse (CIR) was 30.4% overall, 28.7% for allo-SCT, and 37.7% for auto-SCT (*p* = 0.660). Five-year overall survival (OS) was 68.2% for allo-SCT and 64.0% for auto-SCT; progression-free survival (PFS) was 68.2% and 64.0%, respectively (*p* = 0.580, 0.940). Patients with age-adjusted international prognostic index (aaIPI) ≥3 had a significantly higher relapse rate in the auto-SCT group (*p* = 0.022). Univariate analysis identified male sex, aaIPI ≥3, non-CR at cycle 4, and non-CR1 at SCT as adverse prognostic factors. Allo-SCT may benefit patients with high aaIPI.

## 1. INTRODUCTION

T-lymphoblastic lymphoma (T-LBL) is a highly aggressive neoplasm that occurs more frequently in children and young adulthood.^1–4^ The complete remission (CR) rate has been improved to some content by using intensive chemotherapy regimens such as acute lymphoblastic leukemia (ALL)-typed and pediatric-inspired ALL regimens,^5,6^ but the overall survival (OS) rate has not improved significantly. Hu et al^7^ treated 59 T-LBL patients with hyper-CVAD (fractionated cyclophosphamide, vincristine, doxorubicin, and dexamethasone) therapy, the 3-year OS rate was only 37.7%. Moreover, the relapse rate for T-LBL patients who received chemotherapy alone is extremely high, and hardly any salvage regimens were effective for relapsed patients and the 5-year OS rate after relapse was only 14%.^8,9^

Stem cell transplantation (SCT), as the final consolidation treatment of T-LBL, shows a good application prospect.^10–13^ The 5-year OS rate of LBL patients underwent SCT is improved, ranging from 39% to 80%.^14,15^ However, the optimal transplantation strategy, either autologous (auto-) or allogeneic (allo-) SCT, remains unclear. Thus, this retrospective study compared the efficacy and safety of auto-SCT and allo-SCT for T-LBL. We compared the cumulative incidence of relapse (CIR), progression-free survival (PFS), OS, and the outcome of patients, and discussed which kind of SCT could have more advantages for T-LBL patients.

## 2. METHODS

### 2.1. Data collection

This is a retrospective multicenter analysis and medical records of all patients were reviewed for demographics, pretransplant, and transplant therapy-related parameters. The patient population consisted of patients who were diagnosed with T-LBL at 3 centers in China and received either auto-SCT or allo-SCT between January 2008 and December 2018. Patients were included if they were aged between 14 and 60 years; histologically confirmed T-LBL (non-early T-cell precursor ALL/lymphoma populations), in accordance with the World Health Organization (WHO) criteria, as the first primary malignancy at diagnosis; treated with ALL-typed regimen^16^ as induction and consolidation chemotherapy; either had single auto-SCT or allo-SCT. Parameters regarding demographics, pretransplant therapy, transplantation procedures, and outcome data were retrospectively analyzed, with December 2018 as the initial data lock date, and all patients were followed up until the end of December 2023 through medical records and telephone follow-ups.

### 2.2. Definitions

The primary endpoints included CIR and OS. Secondary endpoints included graft-versus-host disease (GvHD) and PFS. According to the 2016 National Comprehensive Cancer Network (NCCN) guidelines and the LUGANO lymphoma response criteria, CR is defined as the normalization of lymph nodes and conglomerate masses, normal lymphoma-related laboratory tests, a post-treatment PET-CT Deauville score of 1 to 3, with or without residual lesions, and, in cases of bone marrow involvement, less than 5% immature lymphoid cells in the bone marrow. A late CR was characterized by the achievement of a response after cycle 2 or the need for mediastinal irradiation. Cumulative incidences of relapse were calculated from the date of transplant to the date of relapse or death, respectively, with the other events being the competing risk. OS was defined as the time between the date of transplant and the date of death or the date of the last follow-up in surviving patients. The PFS was defined as survival in a state of continuous CR.

### 2.3. Statistical analysis

Statistical analyses were performed, and figures were generated using R, version 4.1.2. The measurement data underwent a normality test (Shapiro–Wilk test) and were analyzed using the Mann–Whitney *U* test or independent *t* test as appropriate. Categorical variables, like enumeration data, were analyzed using χ^2^ test or Fisher exact test as appropriate. The correlation between GvHD and relapse was analyzed by the Spearman test. Survival curves were plotted according to the methods of Kaplan and Meier, with differences among them analyzed by the log-rank test. Univariate analysis was performed using a Cox proportional hazards model. A 2-sided *p* < 0.05 indicated a significant difference.

## 3. RESULTS

### 3.1. Patient demographics and treatment characteristics

A total of 69 patients with T-LBL, 16 females and 53 males with a median age of 26 years (range: 16–57), were enrolled in this study. The patients’ demographics and baseline characteristics at diagnosis are detailed in Table 1. No differences in age distribution, sex, age-adjusted international prognostic index (aaIPI), and Eastern Cooperative Oncology Group (ECOG) performance status^17^ were noted between auto-SCT and allo-SCT group. All patients received ALL-typed regimen including VDLP (vincristine, daunorubicin, L-asparaginase, prednisone) in 27 patients, hyper-CVAD (fractionated cyclophosphamide, vincristine, doxorubicin, and dexamethasone) in 27 patients and a modified non-Hodgkin lymphoma Berlin-Frankfurt-Münster-95 (mNHL-BFM-95) regimen in 15 patients.^18^ After 2 to 4 cycles of chemotherapy, 51 patients had CR including 6/51 underwent SCT immediately after 2 cycles of chemotherapy, 12 patients had PR, 3 patients had stable disease (SD), and 3 patients had progressive disease (PD). All patients had 4 or more than 4 cycles of treatment. Patients with PR, SD, or PD achieved CR again after chemotherapy with a modified regimen before SCT.

**Table 1:** Clinical characteristics of patients.

Characteristics	Patients, no. (%)		*p* value
	Auto-SCT	Allo-SCT	
	(n = 25)	(n = 44)	
Age, median (range), y	27 (17–53)	25.5 (15–57)	0.453
Sex			
Male	19	34	0.905
Female	6	10	
Stage			
I/II	5	5	0.331
III/IV	20	39	
aaIPI			
<3	16	18	0.067
≥3	9	26	
ECOG performance status			
0	9	6	0.224
1	5	16	
2	11	22	
Mediastinal involvement	6	10	0.905
Bone marrow involvement	9	30	0.010
Treatment regimens			
VDLP	6	21	<0.001
Hyper-CVAD	6	21	
mNHL-BFM-95	13	2	
Disease status at cycle 4			
CR	17	34	0.403
PR	7	5	
SD	0	3	
PD	1	2	
Time interval from diagnosis to SCT, median (range), mo	9 (6–42)	7 (4–22)	0.214
Disease status at SCT			
CR1	17	33	0.535
non CR1	8	11	

aaIPI = age-adjusted international prognostic index, CR = complete remission, ECOG = Eastern Cooperative Oncology Group, hyper-CVAD = hyper-fractionated administration of cyclophosphamide, vincristine, doxorubicin and dexamethasone, mNHL-BFM-95 = a modified non-Hodgkin lymphoma Berlin-Frankfurt-Münster-95, PD = progressive disease, PR = partial remission, SCT = stem cell transplantation, SD = stable disease, VDLP = vincristine, daunorubicin, L-asparaginase, prednisone.

### 3.2. Patients’ characteristics at transplants

Of the 69 patients who were enrolled in this study, 25 underwent auto-SCT and 44 underwent allo-SCT, including 25 had HLA-matched donors and 19 had haploidentical donors. Thirteen patients received BEAM regimens (carmustine, etoposide, cytarabine, and melphalan), 24 received BUCY plus etoposide regimens (busulfan, cyclophosphamide, and etoposide) and 32 received total body irradiation (TBI, high-energy X-rays, 10 Gy) combined with CTX and etoposide regimens. All patients received CR before SCT. The median time from diagnosis to transplant was 7 months (range 3–42). Median follow-up was 115 months (range 4–134).

### 3.3. Engraftment

The median time to neutrophil engraftment was 12 days (range: 9–30 days) and 10 days (range: 8–25 days) in allo-SCT and auto-SCT group, respectively (*p* = 0.355), and the median time to platelet engraftment was 19 days (range: 9–65 days) and 16 days (range: 9–68 days) in allo-SCT and auto-SCT group, respectively (*p* = 0.691). Chimerism analysis showed that all patients in allo-SCT group achieved full donor chimerism by day +30 after transplantation.

### 3.4. Infection and graft-versus-host disease

Within 100 days after transplantation, 47 patients developed infections, including 13 and 9 cases of bacterial infections, 8 and 4 cases of fungal infections, and 8 and 5 cases of mixed bacterial-fungal infections in the allo-SCT and auto-SCT groups, respectively. The rate of infections was similar between the 2 groups (*p* = 0.376). No significant difference in infection rates was observed between the allo-SCT and auto-SCT groups. The incidence of grade II to IV acute GvHD at day +100 was 45.5% (20/44), with incidences of grade III to IV of 11.4% (5/44). The 2-year incidence of chronic GvHD was 29.6% and no patient had extensive chronic GvHD.

### 3.5. Relapse

**Figure 1.**
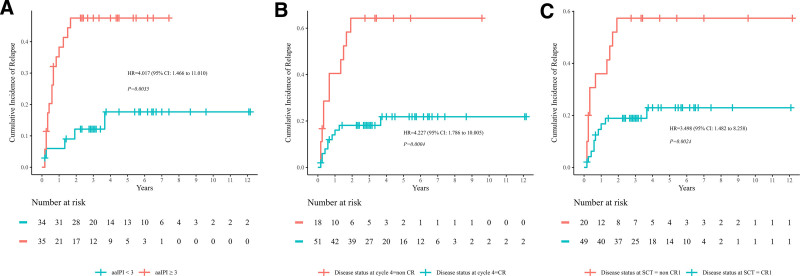
The 5-y CIR in patient subgroups based on adverse prognostic factors. (A) aaIPI: higher CIR in patients with aaIPI ≥3 compared to those with aaIPI <3 (*p* = 0.004). (B) Higher CIR in patients with non-CR at cycle 4 compared to those with CR (*p* = 0.000). (C) Higher CIR in patients with non-CR1 at SCT compared to those with CR1 (*p* = 0.002). aaIPI = age-adjusted international prognostic index, CI = confidence interval, CIR = cumulative incidence of relapse, CR = complete remission, HR = hazard ratio, SCT = stem cell transplantation.

**Figure 2.**
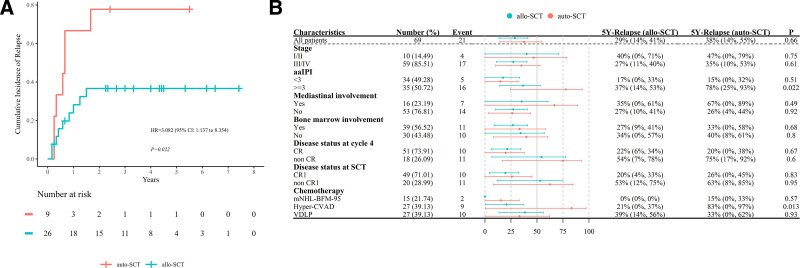
Comparison of 5-Year CIR of patients with aaIPI ≥3 in auto-SCT and allo-SCT. (A) The 5-y CIR in patients with aaIPI ≥3 in auto-SCT was significantly higher than those in allo-SCT group (*p* = 0.022). (B) Subgroup analysis of the 5-y CIR in patients with aaIPI ≥3 receiving auto-SCT or allo-SCT. aaIPI = age-adjusted international prognostic index, CI = confidence interval, CIR = cumulative incidence of relapse, CR = complete remission, CVAD = cyclophosphamide, vincristine, doxorubicin, and dexamethasone, mNHL-BFM-95 = modified non-Hodgkin lymphoma Berlin-Frankfurt-Münster-95, HR = hazard ratio, SCT = stem cell transplantation, VDLP = vincristine, daunorubicin, L-asparaginase, prednisone.

In 69 patients, the 5-year CIR was 30.4% overall. Among these, 21 patients experienced relapsed. The allo-SCT group demonstrated a lower cumulative relapse rate (28.7% [95% confidence interval (CI), 13.5%–41.2%]) compared to auto-SCT group (37.7% [95% CI, 14.3%–54.7%]); however, the difference was not statistically significant (*p* = 0.660). Kaplan and Meier analysis showed that aaIPI ≥3, non-CR at cycle 4, and non-CR1 at SCT were significant adverse prognostic factors for relapse (*p* = 0.004, 0.000, 0.002, **Fig. 1**). The estimated 5-year CIR of patients with aaIPI ≥3, non-CR at cycle 4 and non-CR1 at SCT was 47.5% (95% CI, 27.5%–62.0%), 64.3% (95% CI, 32.3%–81.2%), and 57.3% (95% CI, 28.1%–74.7%), respectively (Table 2). The relapse rate for patients with aaIPI ≥3 was significantly higher (*p* = 0.022) in auto-SCT group vs those in allo-SCT group, suggesting that allo-SCT could provide a potential advantage in reducing relapse rates for patients with aaIPI ≥3 (**Fig. 2A**). All patients had CR before SCT, including those with persisted CR or resisted/relapsed (R/R) patients who achieved CR after salvage therapy. Patients who were not CR1 before auto-SCT or allo-SCT had similar relapse rates after transplantation (62.5% vs 53.1%, *p* = 0.950). The relapse rate of different regimens of chemotherapy before SCT was compared. The 5-year CIR of patients receiving hyper-CVAD as chemotherapy (*p* = 0.013) was lower in allo-SCT group (20.7% [95% CI, 0.3%–37.0%]) vs those in auto-SCT group (83.3% [95% CI, 0.3%–97.2%]). The forest plot is shown in **Figure 2B** showing comparison of 5-year CIR between auto-SCT and allo-SCT stratified by clinical characteristics in patients with T-LBL.

**Table 2:** Univariate analyses of risk factors for relapse, OS, and PFS.

Prognostic factors	Relapse					OS					PFS				
	No.	Probability at 5 y	95% CI		*p* value	No.	Probability at 5 y	95% CI		*p* value	No.	Probability at 5 y	95% CI		*p* value
Age					0.360					0.068					0.180
15–20	14	0.1430	0.000	0.308		14	0.929	0.803	1.000		14	0.929	0.803	1.000	
21–40	51	0.3780	0.218	0.504		51	0.608	0.488	0.758		51	0.608	0.488	0.758	
41–57	4	0.3330	0.000	0.700		4	0.500	0.188	1.000		4	0.500	0.188	1.000	
Sex					0.072					0.042					0.040
Male	53	0.390	0.230	0.516		53	0.604	0.486	0.751		53	0.604	0.486	0.751	
Female	16	0.125	0.000	0.273		16	0.875	0.727	1.000		16	0.875	0.727	1.000	
Stage					0.550					0.310					0.700
I/II	10	0.475	0.000	0.738		10	0.600	0.362	0.995		10	0.600	0.362	0.995	
III/IV	59	0.301	0.170	0.411		59	0.678	0.569	0.808		59	0.678	0.569	0.808	
aaIPI					0.004					0.015					0.004
<3	34	0.176	0.015	0.312		34	0.824	0.705	0.962		34	0.824	0.705	0.962	
≥3	35	0.475	0.275	0.620		35	0.514	0.373	0.710		35	0.514	0.373	0.710	
Mediastinal involvement					0.170					0.018					0.039
Yes	16	0.558	0.099	0.783		16	0.438	0.251	0.763		16	0.438	0.251	0.763	
No	53	0.269	0.138	0.380		53	0.736	0.626	0.865		53	0.736	0.626	0.865	
Bone marrow involvement					0.630					0.210					0.270
Yes	39	0.283	0.126	0.412		39	0.718	0.590	0.874		39	0.718	0.590	0.874	
No	30	0.410	0.146	0.592		30	0.600	0.448	0.804		30	0.600	0.448	0.804	
Disease status at cycle 4					<0.001					0.004					0.001
CR	51	0.218	0.083	0.334		51	0.765	0.657	0.891		51	0.765	0.657	0.891	
Non-CR	18	0.643	0.323	0.812		18	0.389	0.218	0.694		18	0.389	0.218	0.694	
Disease status at SCT					0.002					0.017					0.004
CR1	49	0.229	0.087	0.350		49	0.755	0.644	0.886		49	0.755	0.644	0.886	
Non-CR1	20	0.573	0.281	0.747		20	0.450	0.277	0.731		20	0.450	0.277	0.731	
Type of SCT					0.660					0.580					0.940
Auto-SCT	25	0.377	0.143	0.547		25	0.640	0.477	0.859		25	0.640	0.477	0.859	
Allo-SCT	44	0.287	0.135	0.412		44	0.682	0.557	0.834		44	0.682	0.557	0.834	
Chemotherapy					0.230					0.110					0.130
mNHL-BFM-95	15	0.133	0.000	0.289		15	0.867	0.711	1.000		15	0.867	0.711	1.000	
Hyper-CVAD	27	0.395	0.137	0.576		27	0.556	0.397	0.779		27	0.556	0.397	0.779	
VDLP	27	0.373	0.160	0.531		27	0.667	0.511	0.870		27	0.667	0.511	0.870	
Conditioning regimen					0.250					0.350					0.370
BUCY-based	24	0.292	0.084	0.452		24	0.708	0.548	0.916		24	0.708	0.548	0.916	
TBI-based	32	0.231	0.064	0.368		32	0.719	0.579	0.893		32	0.719	0.579	0.893	
BEAM	13	0.538	0.170	0.743		13	0.462	0.257	0.830		13	0.462	0.257	0.830	

aaIPI = age-adjusted international prognostic index, BEAM = carmustine, etoposide, cytarabine, and melphalan, BUCY = busulfan and cyclophosphamide, CI = confidence interval, CR = complete remission, hyper-CVAD = hyper-fractionated administration of cyclophosphamide, vincristine, doxorubicin and dexamethasone, mNHL-BFM-95 = a modified non-Hodgkin lymphoma Berlin-Frankfurt-Münster-95, OS = overall survival, PFS = progression-free survival, SCT = stem cell transplantation, TBI = total body irradiation, VDLP = vincristine, daunorubicin, L-asparaginase, prednisone.

### 3.6. Survival

**Figure 3.**
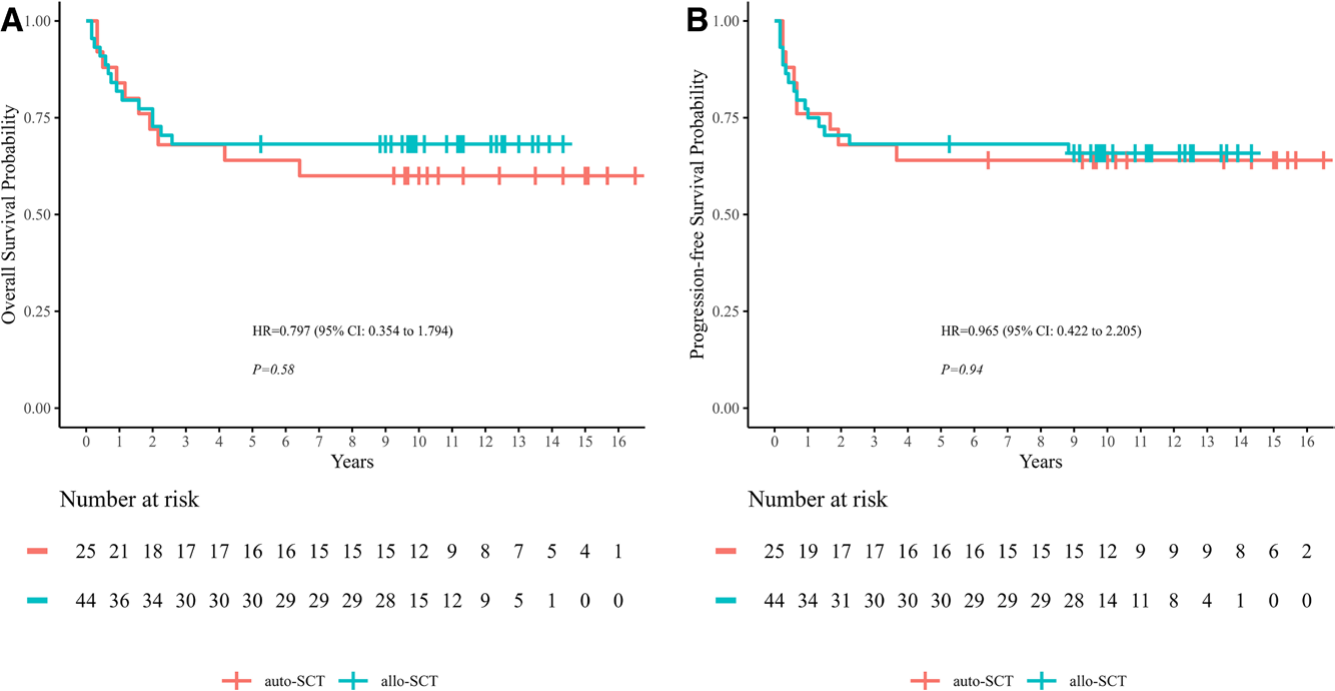
The 5-y OS and PFS rates in auto-SCT vs allo-SCT group. (A) The 5-y OS rate was 68.18% (95% CI, 55.72%–83.43%) in allo-SCT group vs 64% (95% CI, 47.7%–85.9%) in auto-SCT group (*p* = 0.580). (B) The 5-y PFS rate was 68.20% (95% CI, 55.72%–83.43%) in allo-SCT group vs 64% (95% CI, 47.7%–85.9%) in auto-SCT group (*p* = 0.940). CI = confidence interval, HR = hazard ratio, OS = overall survival, PFS = progression-free survival, SCT = stem cell transplantation.

A total of 24 patients died after SCT including 14 patients in allo-SCT group and 10 patients in auto-SCT group. In allo-SCT group, 10 patients died of disease progression and 4 patients died of infection, while in auto-SCT group, 6 patients died of disease progression and 4 patients died of infection. The estimated 5-year OS and PFS rates were 68.2% (95% CI, 55.7%–83.4%) and 68.20% (95% CI, 55.7%–83.4%) in allo-SCT group vs 64% (95% CI, 47.7%–85.9%) and 64% (95% CI, 47.7%–85.9%) in auto-SCT group, with no significant difference between the 2 groups (*p* = 0.580, 0.940). PFS and OS curves are shown in **Figure 3**. The OS curve plateaued 24 months after allo-SCT but was still going down 40 months after auto-SCT. One additional patient died of T-LBL over 50 months after auto-SCT.

**Figure 4.**
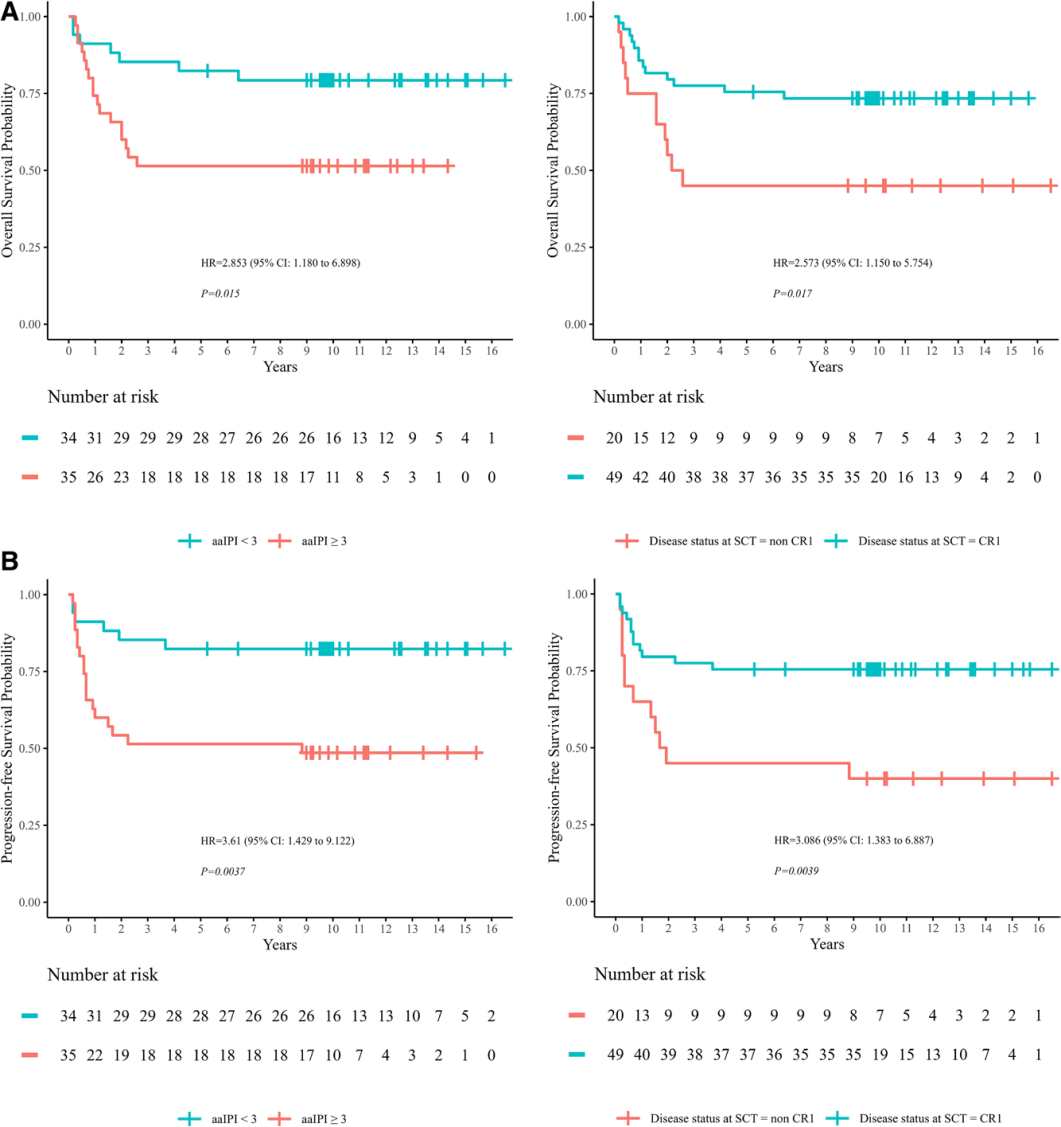
Pretransplant factors in patients were analyzed by univariate analysis and the 5-y OS and PFS in patient subgroups were shown based on adverse prognostic factors (partial display). (A) Higher 5-y OS in patients with aaIPI <3 or CR1 at SCT compared to those with aaIPI ≥3 and non-CR1 at SCT (*p* = 0.015, 0.017). (B) Higher 5-y PFS in patients with aaIPI <3 or CR1 at SCT compared to those with aaIPI ≥3 and non-CR1 at SCT (*p* = 0.004, 0.004). aaIPI = age-adjusted international prognostic index, CI = confidence interval, CR = complete remission, HR = hazard ratio, OS = overall survival, PFS = progression-free survival, SCT = stem cell transplantation.

**Figure 5.**
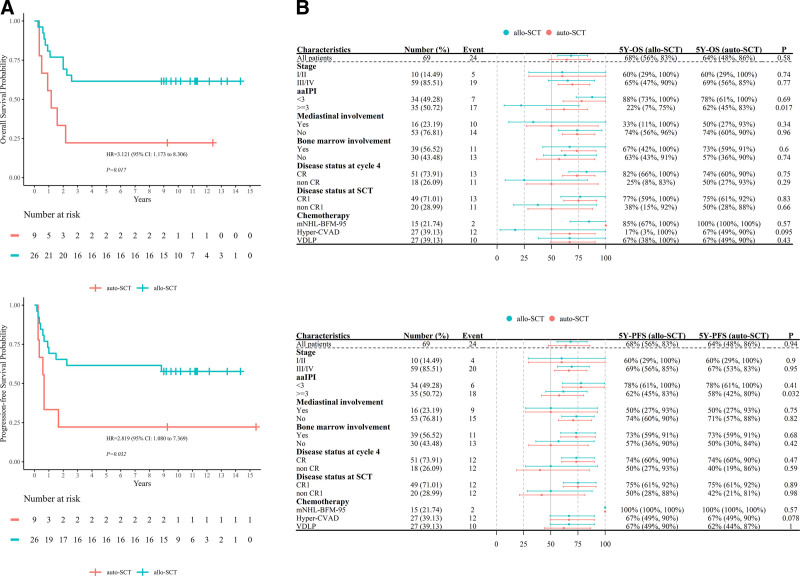
Comparison of 5-Year OS and PFS of patients with aaIPI ≥3 in auto-SCT and allo-SCT. (A) The 5-y OS and PFS rates of patients with aaIPI ≥3 were 61.54% (95% CI, 45.41%–83.39%) and 61.54% (95% CI, 45.41%–83.39%) in allo-SCT group, which were significantly higher than patients in auto-SCT group (22.22% [95% CI, 6.55%–75.44%] and 22.22% [95% CI, 6.55%–75.44%]) (*p* = 0.017, 0.032). (B) Subgroup analysis of the 5-y OS and PFS in patients with aaIPI ≥3 receiving auto-SCT or allo-SCT. aaIPI = age-adjusted international prognostic index, CI = confidence interval, CR = complete remission, CVAD = cyclophosphamide, vincristine, doxorubicin, and dexamethasone, HR = hazard ratio, mNHL-BFM-95 = modified non-Hodgkin lymphoma Berlin-Frankfurt-Münster-95, OS = overall survival, PFS = progression-free survival, SCT = stem cell transplantation, VDLP = vincristine, daunorubicin, L-asparaginase, prednisone.

**Figure 6.**
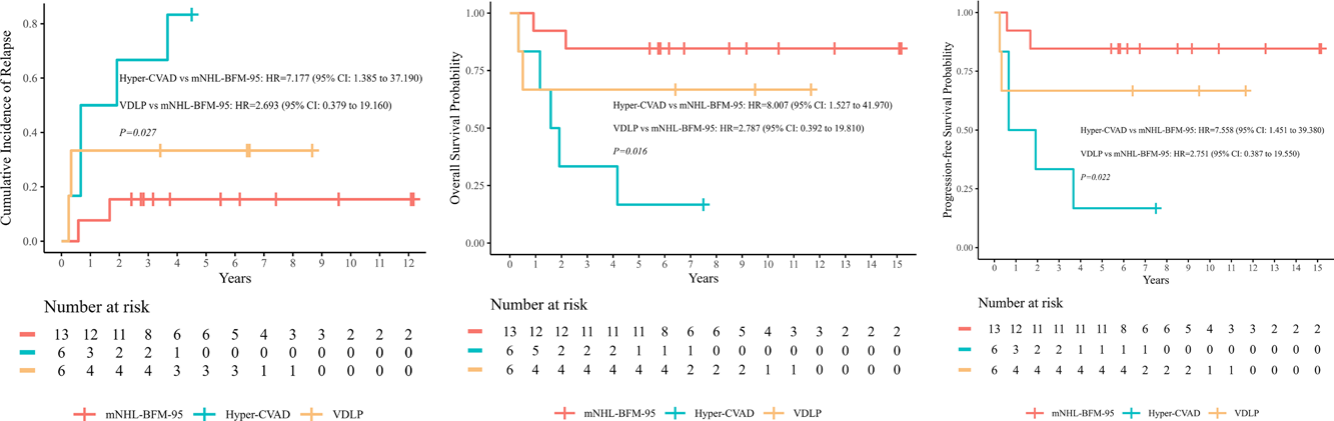
The 5-y CIR, OS, and PFS for patients who received different chemotherapy regimen in auto-SCT group. Patients who received mNHL-BFM-95 demonstrated better 5-y OS (84.6% [95% CI, 67.1–100]), PFS (84.6% [95% CI, 67.1%–100%]), and CIR (15.4% [95% CI, 0%–32.9%]) (*p* = 0.016, 0.022, 0.027). CI = confidence interval, CIR = cumulative incidence of relapse, CVAD = cyclophosphamide, vincristine, doxorubicin, and dexamethasone, HR = hazard ratio, mNHL-BFM-95 = modified non-Hodgkin lymphoma Berlin-Frankfurt-Münster-95, OS = overall survival, PFS = progression-free survival, SCT = stem cell transplantation, VDLP = vincristine, daunorubicin, L-asparaginase, prednisone.

We examined the pretransplant factors in all patients by univariate analysis (Table 2). For both OS and PFS rates at 5 years, male sex, aaIPI ≥3, non-CR at cycle 4 and non-CR1 at SCT were significant adverse prognostic factors (*p* = 0.042, 0.015, 0.004, 0.017; *p* = 0.040, 0.004, 0.001, 0.004, **Fig. 4A, B**). To explore whether allo-SCT could improve OS or PFS for patients with pretransplant adverse factors, subgroup analyses were performed according to transplant types. The 5-year OS and PFS rates of patients with aaIPI ≥3 were 61.5% (95% CI, 45.4%–83.4%) and 61.5% (95% CI, 45.4%–83.4%) in allo-SCT group, which were significantly higher than patients in auto-SCT group (22.2% [95% CI, 6.6%–75.4%] and 22.2% [95% CI, 6.6%–75.4%]) (*p* = 0.017, 0.032, **Fig. 5A**). The estimated 5-year OS rate of the patients who were non-CR at cycle 4 and non-CR1 at SCT, were 50.0% and 50.0% in allo-SCT group, vs 25.0% and 37.5% in auto-SCT group, respectively, and the difference was not statistically significant. Additionally, we observed that mediastinum involvement was another adverse factor for OS (*p* = 0.018), with the OS rates of 43.8% (95% CI, 25.1%–76.3%), much worse than those without mediastinum involvement (73.6% [95% CI, 62.6%–86.5%]). Different conditioning regimens were compared to determine the optimal one for SCT, however, comparison of 5-year OS and PFS in patients under different conditioning regimens revealed no statistically significant differences (*p* = 0.350, 0.370). Forest plot is shown in Figure 5B showing a comparison of 5-year OS and PFS between auto-SCT and allo-SCT stratified by clinical characteristics. Given that the benefits of auto-SCT compared to allo-SCT are not significantly different for many patients, it becomes crucial to explore how to improve their post-transplant survival within the auto-SCT framework. We evaluated the impact of chemotherapy regimens within the auto-SCT group. It was observed that patients who received mNHL-BFM-95 demonstrated significantly better 5-year OS (84.6% [95% CI, 67.1%–100%]), PFS (84.6% [95% CI, 67.1%–100%]), and CIR (15.4% [95% CI, 0%–32.9%]) compared to those who received hyper-CVAD or VDLP (*p* = 0.012, 0.022, 0.027, **Fig. 6**).

In allo-SCT group, 5-year OS and PFS rates of patients with GVHD I/II were 72.5% (95% CI, 59.9%–87.8%) and 72.5% (95% CI, 59.9%–87.8%), whereas those with grade III/IV acute GvHD were only 25.0% (95% CI, 4.6%–100%) and 25.0% (95% CI, 4.6%–100%), respectively (*p* = 0.048, 0.041). Severe GvHD has attributed to lower OS and PFS rates but GVL effects have not been preserved, resulting in similar relapse rate of patients after allo- and auto-SCT.

## 4. DISCUSSION

High recurrence rates remain the most significant problem of T-LBL.^4,19^ A key therapeutic issue is what advanced approaches after CR should be used in prolonging remissions and long-term survival. Both auto-SCT and allo-SCT are used in an attempt to reduce relapse rates, but it is not known which option represents the better therapy for patients with T-LBL. In this study, we found that allo-SCT demonstrates potential superiority in 5-year OS, PFS, and CIR for patients with aaIPI ≥3 when compared to auto-SCT. However, this finding should be interpreted with caution due to the small sample size in subgroup analysis. There were no significant differences between auto-SCT and allo-SCT concerning 5-year OS, PFS, and CIR for patients with mediastinal or bone marrow involvement, and with resisted/relapsed disease, but allo-SCT still showed a positive but non-statistically significant trend in association with improving relapse.

Few studies of auto-SCT in T-LBL reported dismal outcomes. In a total of 2216 patients with T-LBL who underwent SCT between 1991 and 2017 registered in the TRUMP database, 27 patients underwent auto-SCT, 1 received syngeneic SCT, and 2188 were treated with allo-SCT. Patients who received auto-SCT had poor outcomes, with a 1-year OS rate of 18.5% and a median OS time of 5.6 months.^20^ All 27 patients died during the observation period and 71.4% died of disease relapse and progression.^20^ Allo-SCT was subsequently performed since 1987 showing promising results.^8,14,21,22^ Fukushima et al^23^ reported estimated 3-year OS, PFS, and relapse-free survival of 45.3%, 39.3%, and 33.8%, respectively, for T-LBL patients underwent allo-SCT. More and more results about comparison of allo-SCT and auto-SCT for T-LBL were reported. In Levine’s study, T-LBL patients had a 3-year CIR of 34% after allo-SCT, which was lower than that of 52% in auto-SCT group, but the 5-year adjusted PFS were similar between allo-SCT and auto-SCT (36% vs 39%).^14^ In a retrospective study of 41 T-LBL patients receiving auto-SCT, 3-year CIR was (30.7 ± 7.4) % which was similar to that of allo-SCT in Levine’s report.^24^ Not surprisingly, in the present study, auto-SCT has shown a 5-year CIR, OS, and PFS of 37.70%, 64%, and 64%, respectively, suggesting relatively promising results as previously reported, except patients with aaIPI ≥3. Therefore, for patients with aaIPI <3, it is essential to investigate ways to further enhance the post-transplant survival within the auto-SCT setting. We compared the clinical outcome of patients treated with different chemotherapy regimen in auto-SCT group and found that mNHL-BFM-95 regimen might offer a more effective therapeutic approach. The absence of statistical significance in some comparisons may partly be attributed to sample size limitations and variability in patient characteristics, highlighting the need for further studies to confirm these findings.

Would allo-SCT significantly improve clinical outcome for patients with aaIPI ≥3 and other specific risk factors? Allo-SCT showed superiority in 5-year OS, PFS, and CIR for patients with aaIPI ≥3 in this study. Older age, male sex, poor performance status, and non-CR at the time of SCT were reported to be prognostic factors for poor outcomes.^12,14,25–27^ Our study findings are consistent with these reports but should be interpreted cautiously given the inherent limitations of subgroup analysis in small patient cohorts. Except for male and non-CR1 at SCT, that needing over 4 cycles to achieve CR which indicated less sensitivity to chemotherapy was another independent risk factor for relapse, PFS, and OS. Mediastinal involvement was one of the adverse factors for OS though patients with residual mediastinal mass received consolidation radiotherapy. The mediastinal recurrence rate was 7.25% in our study which was similar to 5% to 10% in reported cases without prevention or consolidation radiotherapy,^7,28^ suggesting that radiotherapy did not appear to improve mediastinal relapse. Regarding the conditioning regimen, Niu et al^29^ reported that the 2-year CIR and OS in TBI-based group were 9.1% and 83%, which showed significant improvement as compared with that of 49.6% and 35% in BU-based group. TBI-based conditioning regimen might be an optimal choice for adult patients with T-LBL undergoing allo-HSCT. However, in our study, neither TBI- nor BU-based regimen showed significant superiority in improving CIR, OS, nor PFS in the allo-HSCT group. Comparison of 5-year OS and PFS in patients under different conditioning regimens revealed no statistically significant differences.

Allo-SCT had inferior PFS for the first 6 months following transplantation and superior PFS after 6 months, which suggested that survival disadvantage with allo-SCT through the first 6 months could be attributed to uncontrolled acute GvHD.^14,25,30–34^ The prognoses of patients with mild acute GvHD were superior to those with severe acute GvHD, and those without acute GvHD as well.^12,28^ However, studies did not show any benefit of GvHD on the prevention of relapse, which remains the main cause of treatment failure.^33^ In our study, both allo- and auto-SCT had similar OS and PFS, and grade III/IV acute GvHD was associated with worse outcome as reported, which implied that severe GvHD was not associated with significant GVL effect but only GvHD-induced organ damage. Therefore, developing strategies to reduce GvHD severity while strengthening the GVL effect is a key area for future research. It is reported that GVL effect induced by the cessation of immunosuppressant could be more significant and played a critical role in the reduction of tumor burden even when T-LBL patients relapsed post-SCT.^12,25,28,33^ Whether GVL effect should be strengthened early after allo-SCT by immunosuppressant withdrawn or donor lymphocyte infusion to T-LBL patients especially those with adverse factors, warrants further investigation.

## 5. CONCLUSIONS

For patients with aaIPI <3, where overall clinical outcomes between auto-SCT and allo-SCT are comparable, enhancing auto-SCT outcomes becomes pivotal, and mNHL-BFM-95 chemotherapy may enhance clinical outcomes in auto-SCT recipients. Conversely, for patients with aaIPI ≥3, allo-SCT demonstrates superiority in improving transplantation outcomes, underscoring its importance in this high-risk subgroup.

## ACKNOWLEDGMENTS

We would like to thank all personnel at the Hematology Department, The Fifth Affiliated Hospital of Guangzhou Medical University for their support during the project. The authors declare that artificial intelligence is not used in this study.

## ETHICAL APPROVAL

The study was approved by the ethics committee of Guangzhou Medical University, Guangzhou, China (Project title: Advanced Therapeutic Strategies in T-Cell Lymphoma/leukemia and related hemophagocytic lymphohistiocytosis; Reference No: GYWY-K2024-07; date of approval: January 3, 2024). The patients legally authorized representative provided written informed consent for participation in the study.

## AUTHOR CONTRIBUTIONS

X.L.: Conceptualization; formal analysis; methodology; software; writing - original draft; prepared figures. Z.F.: Investigation; formal analysis; and prepared Table 1. R.Z.: Investigation; data curation; prepared Table 2. F.H. and N.X.: Data collection. P.Q., J.L., and C.W.: Investigation; prepared Table 1; and data curation. Q.L., H.T., and H.H.: Project administration; validation; writing - review & editing; supervision.
